# Consequences of a global enzyme shortage of agalsidase beta in adult Dutch Fabry patients

**DOI:** 10.1186/1750-1172-6-69

**Published:** 2011-10-31

**Authors:** Bouwien E Smid, Saskia M Rombach, Johannes MFG Aerts, Symen Kuiper, Mina Mirzaian, Hermen S Overkleeft, Ben JHM Poorthuis, Carla EM Hollak, Johanna EM Groener, Gabor E Linthorst

**Affiliations:** 1 Department of Internal Medicine, Division of Endocrinology and Metabolism, Academic Medical Centre, PO Box 22660, 1100 DD, Amsterdam, The Netherlands; 2 Department of Medical Biochemistry, Academic Medical Centre, PO Box 22660, 1100 DD, Amsterdam, The Netherlands; 3 Bio-organic Synthesis, Leiden Institute of Chemistry, Leiden, The Netherlands

## Abstract

**Background:**

Enzyme replacement therapy is currently the only approved therapy for Fabry disease. From June 2009 on, viral contamination of Genzyme's production facility resulted in a worldwide shortage of agalsidase beta leading to involuntary dose reductions (approved dose 1 mg/kg/eow, reduced dose 0.5 mg/kg/m), or switch to agalsidase alpha (administered dose 0.2 mg/kg/eow). An assessment report from the European Medicines Agency (EMA) raised serious concerns about an increase in adverse events at lower dosages of agalsidase beta. We determined the influence of the shortage on clinical event incidence and the most sensitive biochemical marker (lysoGb3) in Dutch Fabry patients.

**Methods:**

The incidence of clinical events per person per year was calculated from start of agalsidase beta treatment until the shortage, and was compared to the incidence of clinical events during the shortage period. In addition, plasma lysoGb3, eGFR, quality of life (SF-36) and brief pain inventory (BPI) questionnaires were analysed.

**Results:**

All thirty-five Dutch Fabry patients using agalsidase beta (17 males) were included. Mean clinical event incidence was unchanged: 0.15 events per person per year before versus 0.15 during the shortage (p = 0.68). In total 28 clinical events occurred in 14 patients during 4.6 treatment years, compared to 7 events in 6 patients during the 1.3 year shortage period. eGFR and BPI scores were not significantly altered. Two SF-36 subscales were significantly but minimally reduced in females. In males, lysoGb3 increased with a median of 8.1 nM (range 2.5 - 29.2) after 1 year of shortage (p = 0.001). Increases in lysoGb3 were found in both patients switching to agalsidase alpha and on a reduced agalsidase beta dose. Antibody status, treatment duration or clinical event incidence showed no clear correlation to lysoGb3 increases.

**Conclusions:**

No increase in clinical event incidence was found in the adult Dutch Fabry cohort during the agalsidase beta shortage. Increases in lysoGb3, however, suggest recurrence of disease activity.

## Background

Fabry disease (OMIM 301500) is a rare inherited X-linked lysosomal storage disease. Mutations in the *GLA *gene cause a deficiency of the lysosomal enzyme α-galactosidase A. As a result glycosphingolipids with a terminal α-galactosyl moiety, predominately globotriaosylceramide (Gb3), accumulate in lysosomes [1]. This accumulation is believed to result in the symptoms and complications of the disease. During childhood presenting symptoms consist of characteristic neuronopathic pains, gastro-intestinal complaints and hypohidrosis. Complications usually occur later in life and include progressive renal insufficiency, stroke, cardiac hypertrophy or infarction, and cardiac arrhythmia [2]. The phenotype of the disease is very variable, ranging from severe end-organ damage and early death in classically affected males to less pronounced disease manifestations in some male and the majority of female patients.

Enzyme replacement therapy (ERT) is currently the only approved therapy for Fabry disease and aims at restoring the defective degradation of accumulated substrates by infusion of recombinant α-galactosidase A. In 2001 the European Medicines Agency (EMA) approved two recombinant enzyme preparations in Europe: agalsidase alpha (Replagal™, Shire, at a registered dose of 0.2 mg/kg/eow) and agalsidase beta (Fabrazyme, Genzyme, at a registered dose of 1.0 mg/kg/eow). In the USA, only agalsidase beta is licensed. Treatment with both preparations is reported to diminish Gb3 in tissue biopsies, decrease left ventricular hypertrophy and stabilize renal function [3-8]. These effects seem most prominent in patients with less severe organ involvement at start of therapy [9,10]. Studies on the effect of ERT on the prevention of Fabry related complications are limited. One phase IV study was conducted showing limited efficacy of treatment with agalsidase beta showing a modest decrease in incidence of complications [9]. Such a study was never performed for agalsidase alpha. Although one study could not demonstrate differences between agalsidase alpha and agalsidase beta at and equal dose of 0.2 mg/kg/eow [11], the superiority of either one of the products at their registered dose has not been proven so far.

In June 2009 Genzyme identified a virus (vesivirus 2117) in one of the six bioreactors at their Allston manufacturing facility. Genzyme has reported that this virus is not known to cause disease in humans. Genzyme temporarily interrupted its production, which resulted in a worldwide shortage of agalsidase beta. Assuming a quick recovery of full production, the EMA advised in an online press release on 25 June 2009 [12] that: *' priority should be given to children, adolescent, and adult male patients. However, adult female patients in whom the disease is less severe may receive Fabrazyme at a reduced dose'*. This decision was based upon the supposition that female patients with no clinically significant end organ damage could more easily tolerate a lower dose of therapy. In a subsequent online press release on 23 April 2010 [13] the EMA advised that: ' *for patients on the reduced dose who demonstrate a deterioration of the disease, physicians should consider restarting the original treatment with the full dose of Fabrazyme or switching to an alternative treatment, such as Replagal*'. Initially, in the Netherlands only less affected female patients received a reduced dose. However, from October 2009 persisting shortages forced dose reductions in all Dutch patients, including males. In the absence of sufficient agalsidase beta to restore full dose, Dutch Fabry patients could indicate their preference to stay at a reduced agalsidase beta dose or switch to agalsidase alpha.

On 19 October 2010 the EMA released an assessment report on the shortage of agalsidase beta noting an increase in reporting of adverse events since the start of the shortage, possibly due to the lowered dose [14]. More specifically, it was stated that: '*this pattern of adverse events resembles the natural, but accelerated, course of Fabry disease*'. In addition, the post-marketing registry on outcomes of treatment with agalsidase beta (the Fabry Registry, sponsored by Genzyme) showed that a higher percentage of reports was received of patients suffering from neuronopathic pains, diarrhoea and abdominal pain, compared to the period before the shortage[14]. This suggested increase in adverse events and complaints is difficult to interpret. It is possible that indeed a lower dose of agalsidase beta leads to disease progression or to an accelerated disease course. However, it is also possible that the anxiety caused by the shortage and the recommendations by the EMA to treat patients at full dose of agalsidase beta in case of an adverse event, led to increased awareness and reporting of adverse events. Thus, there is a need for objective data to assess the impact of the agalsidase beta shortage. We studied the incidence of clinical events per person per year before and during the shortage in the adult Dutch Fabry cohort. In addition, we analyzed lysoGb3, eGFR, quality of life (SF-36) and brief pain inventory (BPI) questionnaires.

## Methods

### Patients

All adult Dutch Fabry patients treated with agalsidase beta before the shortage were included in this retrospective observational study (n = 35). Diagnosis was previously confirmed by deficient α-galactosidase A activity (males) and genotyping (males and females). As part of the standard clinical practice, most patients are routinely seen at the outpatient clinic every 3 months for recording of complaints and clinical events, physical examination, and plasma creatinine analysis. In addition, 24-h urine collections, BPI (Brief Pain Inventory) and SF-36 questionnaires, cardiac ultrasound, electrocardiogram, hearing tests, and brain imaging are performed on a yearly basis. All these evaluations were continued during the shortage. The hospital's Ethical Committee of the AMC reviewed the protocol and deemed it as being a non-interventional study, which does not require formal approval under Dutch law.

### Disease progression criteria

Progression of disease was classified as a disease associated event according to the following slightly amended criteria, which were previously described [9,11], or death. A neurological event was defined as a stroke or transient ischemic attack as diagnosed by a neurologist. A renal event was defined as progression of renal disease to CKD stage 5 (MDRD < 15 ml/min/1.73 m^2^, kidney transplantation, dialysis) or a 33% increase in plasma creatinine on 2 consecutive time points. A cardiac event was defined as symptomatic cardiac arrhythmia requiring anti-arrhythmic medication, implantation of an ICD or pacemaker, hospitalization for heart failure or cardiac arrhythmia, myocardial infarction, coronary artery bypass graft (CABG) or percutaneous transluminal coronary angioplasty (PTCA). If symptomatic cardiac arrhythmia coincided with another cardiac event such as myocardial infarction or hospitalization for cardiac arrhythmia only one single clinical event was scored. The clinical event incidence per year per person was calculated from start of agalsidase beta treatment until the time of shortage and was compared to the clinical event incidence per year per person during the agalsidase beta shortage. Since clinical event incidence may increase as a result of aging, there is a potential risk of overestimating the event risk after the shortage when comparing it to the entire pre-shortage treatment period. In order to minimize this risk, an equal period of time directly before the shortage was compared to the period after the shortage.

### Additional clinical evaluations

Decline of glomerular filtration rate (eGFR) was estimated by the abbreviated MDRD [15,16] and was compared for an equal period of time before and during the shortage. Left ventricular mass was assessed by cardiac ultrasounds using the Devereux formula indexed for height [17]. Quality of life (QOL) was assessed using the SF-36 questionnaire [18]. The SF-36 is a generic questionnaire with 36 items that measures functional health and well-being. It comprises eight domains: physical functioning, role physical, bodily pain, general health, vitality, social functioning, role emotional, mental health, and two summary components (consensus on the reliability of the summary components has not yet been achieved (e.g. [19,20]). Domain and summary component scores range from 0-100; higher scores correspond to better health status or well-being. Minimal important clinical improvement (MCID) on the SF-36 subscales, defined as:' *the smallest difference in score in the domain of interest which patients perceive as beneficial or harmful and which would mandate, in the absence of troublesome side effects and excessive costs, a change in the patient's health (health care) management*' [21], has never been determined in Fabry disease. Therefore, MCID from other chronic illnesses were used for comparison [22,23]. Assessment of pain was performed using the BPI questionnaire. Average BPI and SF-36 scores were calculated for the period of the shortage and an equal period before the shortage and subsequently compared.

### Biochemistry

Lysoglobotriaosylceramide (lysoGb3) levels in plasma were measured with a newly developed method based on tandem mass spectrometry with isotope labelled lysoGb3 as internal standard (manuscript in preparation). Plasma lysoGb3 was investigated in all males and females who were treated for at least one year with ERT before start of shortage (since lysoGb3 is known to stabilize thereafter [24]) and with an increased lysoGb3 at baseline. Lipid concentrations of two plasma samples before and two during the shortage were available, all on slightly different time points. Baseline MSSI (Mainz severity score index[25]) scores were used to assess differences between patients experiencing increase in lysoGb3 or clinical events. Antibody (AB) analysis was performed by incubating blood of patients with 1 ng agalsidase beta. The amount of plasma needed to neutralize 50% of the enzymatic activity of 1 ng agalsidase beta was determined by titration. A sample was determined not neutralizing when a maximum plasma input of 15 μl (with a final volume of 200 μl) caused less than 50% neutralization.

### Statistical analysis

Statistical analyses were performed using SPSS 16.0 (IBM, Chicago). Continuous data are expressed as median (range), or mean (SD) when appropriate. Frequencies of clinical events during the shortage between treatment groups were compared using Fisher exact test. Differences in clinical event incidence per person per year and differences in lysoGb3, left ventricular mass, SF-36 and BPI scores were analysed using paired Wilcoxon signed rank test. To analyse decline of eGFR repeated measures were performed using a linear mixed model. The intercept was included in the random model as well as the follow-up, thereby optimizing the Akaike Information criterion. Covariates included follow-up and the interaction term for follow-up and the shortage period as a dichotomous variable. Spearman rank tests were used to assess correlations between lysoGb3 and clinical response. Two tailed p-values < 0.05 were considered significant.

## Results

### Patient characteristics

**Figure 1:**
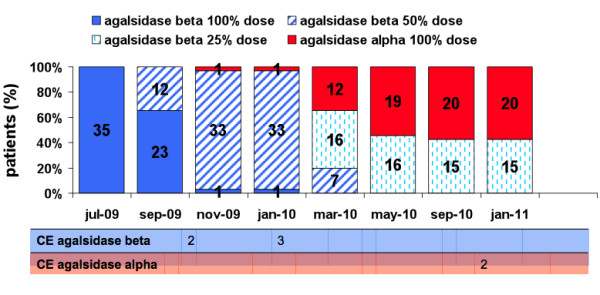
**Course of preparations and dosages during agalsidase beta shortage**. The numbers in the bars represent the number of patients in different dosage and preparation groups being the *continued agalsidase beta group*, the *direct *and *later switch *to agalsidase alpha group. The occurrence of clinical events during agalsidase dose reduction or during switch to agalsidase alpha is outlined on a schematic timeline below the figure. Abbreviations: CE: clinical events.'

Thirty-five Fabry patients (17 males) were included in this study. Median age was 51 years (range: 18-69), with a median treatment duration until the shortage of 5.4 years (range: 3 months-10.3 yr). The median shortage duration for all patients was 1.3 (range 0.9-1.5) years. Patients were divided in three treatment groups: an *immediate switch *group, a *later switch *group and a *continued agalsidase beta *group (Figure 1). The *immediate switch *group consisted of patients who directly switched to agalsidase alpha at the start of the agalsidase beta shortage (n = 2). The *later switch *group consisted of patients who first had a dose reduction of agalsidase beta (from 1.0 mg/kg/eow to 0.5 mg/eow or 0.5 mg/kg/month) and subsequently switched to agalsidase alpha (n = 18). Finally, the *continued agalsidase beta *group consisted of patients who continued on their reduced dose: from 1.0 mg/kg/eow to 0.5 mg/kg/month (n = 15). The median treatment duration with agalsidase alpha in the *immediate *and *later switch *group was 0.9 year (range: 0.5-1.4 yr.).

### Clinical event incidence during the agalsidase beta shortage

During the agalsidase beta shortage 7 clinical events occurred. Five events occurred in patients while their agalsidase beta dose was reduced, whereas two clinical events took place after the switch to agalsidase alpha (see Figure 1). Events included a stroke, an implantation of a pacemaker because of total atrio-ventricular-block, 1 scheduled implantation of a cardiac defibrillator due to a non-sustained ventricular tachycardia already present before the agalsidase beta shortage, 1 kidney transplantation due to pre-existent chronic kidney failure, 2 hospitalizations because of cardiac arrhythmia, and 1 myocardial infarction. The patients in whom a clinical event occurred during the shortage were significantly older: 55.3 vs. 46.7(p = 0.02) compared to those not having a clinical event, but their median treatment duration or baseline MSSI did not differ significantly (p = 0.79, respectively p = 0.15). The number of clinical events which occurred during the agalsidase beta dose reduction or during the switch to agalsidase alpha did not differ significantly (p = 0.11).

The median clinical event incidence per person per year did not differ significantly (p = 0.68), neither for males and females separately (table 1). When the clinical event incidence was calculated for an equal period of time before as during the shortage, no differences in clinical event incidence were found (male p = 0.25, females p = 0.14). To compare agalsidase beta 1.0 mg/kg/eow with the period of agalsidase beta dose reduction, clinical event incidence was determined until switch to agalsidase alpha. Again, no significant differences were found in clinical event incidence (p = 0.77).

**Table 1:** Clinical event incidence before and during shortage.

	Before shortage					During shortage					
	CE n	Mean follow-up	Mean CE incidence	Median CE incidence	Range	CE n	Mean follow-up	Mean CE incidence	Median CE incidence	Range	P
Male (n = 17)	20	5.3	0.23	0.17	0-1	3	1.32	0.14	0	0-1.62	0.25
Female (n = 18)	8	3.9	0.07	0	0-0.68	4	1.32	0.16	0	0-0.83	0.40
Total	28	4.6	0.15	0	0-1	7	1.32	0.15	0	0-1.62	0.68

Clinical event (CE) incidence (clinical events per person per year) was compared before and during shortage.

### eGFR

Two patients were excluded from the analysis: one patient received a kidney transplant shortly before the onset of shortage, of the other patient no creatinine value before the shortage was available. The percentage of patients using ACE-inhibitors or AT2-antagonist (64%) during the study was stable and comparable between groups. Mean MDRD decline per year before and during the shortage did not differ significantly for males; before: - 0.81 mL/min/1.73 m^2^/year (CI: -5, 3.4), during: -1.12 (CI:-2.6, 2.0), p = 0.79, or for females; before: -1.12 (CI: -5.6, 3.3), during: -1.38 (CI:-3.7, 3.20), p = 0.88.

### Left ventricular mass

Analysis of left ventricular mass was restricted due to limited data. Only 12 patients (3 males) had a cardiac ultrasound available after one year of the onset of the shortage. The change in left ventricular mass in these patients ranged from -13 to + 38 gram/m^2.7 ^(p = 0.9) and showed no correlation to clinical event incidence, age or plasma lysoGb3.

### *Pain assessment using BPI *questionnaires

BPI scores before and during the shortage were available for twenty-nine patients, with a median number of five questionnaires (range: 3-8). No statistical differences were found for any of the BPI items before and during the shortage. Patients for whom BPI was not available did not report an increase of pain during the shortage.

### Quality of Life, SF-36

**Figure 2:**
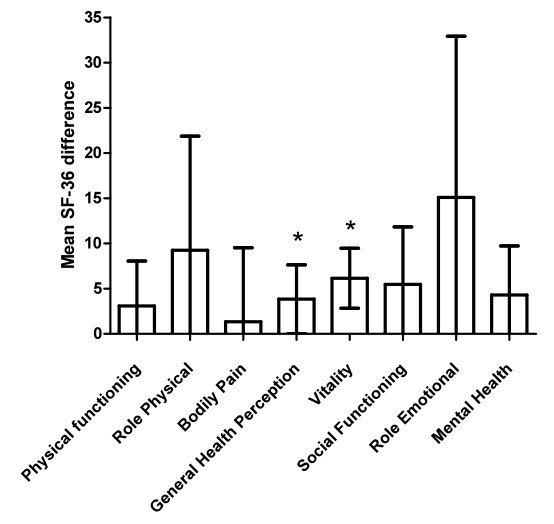
**Mean difference SF-36-scores in females**. Error bars represent 95% confidence intervals. An asterisk represents statistical significance of p < 0.05.

For thirty patients SF-36 questionnaires were available before and during the shortage, with a median number of five questionnaires (range 3-8). Though almost all scales decreased during the shortage, only statistical significant differences were found in females for the general health perception and vitality subscale (table 2 and Figure 2). Comparing our mean changes to the minimal clinical important difference determined in other chronic diseases (general health subscale: 2.4 vitality: 5-7.8) [21-23], the changes were on the borderline of clinical meaningful differences.

**Table 2:** SF-36 subscale scores before and during shortage

	Before Shortage		During Shortage					
**Male **(n = 14)	Mean	SD	Mean	SD	Mean difference	CI (95%) Mean difference		p
						Upper	Lower	
Physical functioning	60.0	30.1	59.6	26.5	0.4	-9.5	103	0.75
Role physical	43.2	40.4	32.4	35.3	10.7	-11.9	33.2	0.24
Bodily pain	64.3	24.9	60.1	19.2	4.2	-7.7	16.1	0.53
General health	31.2	17.4	36.5	24.8	-5.4	-14	3.2	0.34
Vitality	50.2	17.5	50.0	19.6	0.12	-7.5	7.7	0.7
Social functioning	66.5	30.3	59.8	21.9	6.7	-4	17.4	0.18
Role emotional	57.1	51.4	66.3	39.6	-9.1	-29.5	11.3	0.4
Mental health	74.2	17.4	78.8	13.3	-4.6	-12.1	2.8	0.25

	**Before Shortage**		**During shortage**					

**Female **(n = 16)	Mean	SD	Mean	SD	Mean difference	CI (95%) Mean difference		p
						Upper	Lower	
Physical functioning	61.5	26.3	58.4	24.8	3.1	-1.9	8.1	0.10
Role physical	36.8	39.8	27.6	40.2	9.2	-3.4	21.9	0.11
Bodily pain	62.3	23.7	61.0	23.5	1.3	-6.8	9.5	0.74
General health	41.4	18.9	37.6	20.9	3.8	0	7.6	0.03
Vitality	42.7	22.1	36.5	23.1	6.1	2.8	9.5	0.003
Social functioning	68.5	18.6	63.1	27.3	5.5	-0.9	11.8	0.11
Role emotional	72.4	34.5	57.3	35.5	15.1	-2.7	32.9	0.15
Mental health	70.7	15.6	66.4	19.6	4.3	-1.1	9.7	0.10

SF-36 scores from females and males separately before and during the shortage. P-values represent SF-36 scores compared before and during the shortage.

### Spontaneous reported complaints during agalsidase beta shortage

Twenty-three patients reported complaints spontaneously at our outpatient clinic. They consisted of: tiredness (n = 11), gastro-intestinal complaints (n = 3, pre-existing), increase of angiokeratomas (n = 1), palpitations (n = 3, pre-existing), collapse e.c.i (n = 2, no cardiac arrhythmia found), angina (n = 5, no proven ischemia by cardiologist), dyspnoea (n = 2, one new complaint), and headache (n = 1, pre-existing). Three patients experienced neurononopathic pains, of which one patient reported pain for the first time. The other two had no aggravation of previous existing pain.

### Plasma lysoGb3

**Figure 3:**
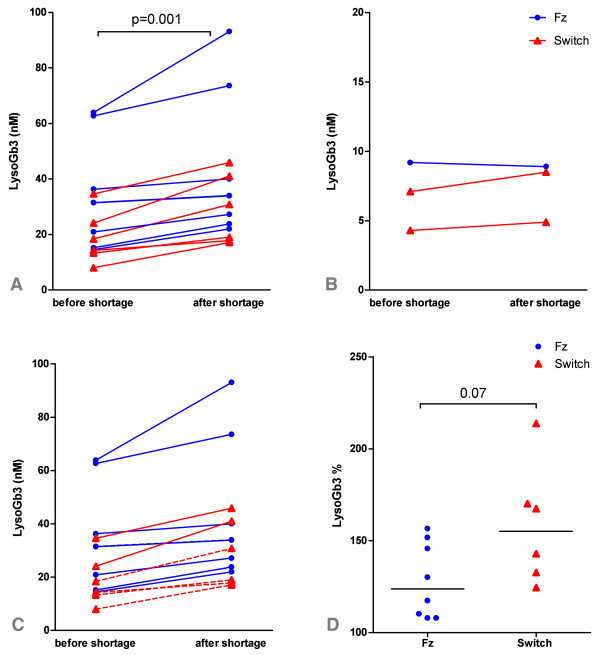
**Individual plasma lysoGb3 levels before and after approximately 1 year of shortage for 3a**. males and **3b**. females. In males a significant increase of lysoGb3 is seen after 1 year of shortage. **3c**. Males with (AB+, solid line) and without antibodies (AB-, dashed line) do not show a significant difference in lysoGb3 increase. **3d**. Percentage increase of lysoGb3 after 1 year of shortage in the *continued agalsidase group *vs. *later switch *group (males only). The *continued agalsidase group *was dose reduced from 0.5 mg to 0.25 mg/kg/eow. The *later switch *group was first dose reduced to 0.5 mg/kg/eow agalsidase beta and subsequently switched tot agalsidase alpha 0.2 mg/kg/eow. In the *continued agalsidase beta *group median lysoGb3 value at start of shortage (= 100%) was 31.5 nM and 16.4 nM in the *switch *group. Abbreviations: Fz: *continued agalsidase beta *group (blue line), switch: *later switch *group (red line).

Plasma lyso Gb3 was determined in a subset of 17 patients (14 males). In males, median lysoGb3 before the shortage was 22.5 nM (range: 8-63.9) and 32.4 nM (range: 17.1-93.1) after ± 1 year of shortage. A statistical significant increase of 8.1 nM (range: 2.5-29.2) lysoGb3 was seen after approximately 1 year of the shortage (p = 0.001) (see Figure 3a). For females, median lysoGb3 before the shortage was 7.1 nM (range: 4.3-9.2) and 8.5 nM (range: 4.9-8.9) after approximately 1 year of shortage, which was not statistically different (p = 0.3) (see Figure 3b). The increase in lysoGb3 in men was significantly larger than in females (8.1 vs. 0.6 nM, p = 0.003). Values did not return to pre-treatment values (data not shown). Increase in lysoGb3 was found both in patients who switched to agalsidase alpha and patients on a reduced agalsidase beta dose (7.4 vs. 6.3 nM p = 0.7). These groups were differed in respect to lysoGb3 levels in the pre-shortage samples, which tended to be higher in the *reduced agalsidase beta *group (31.5 vs. 16.4 nM, p = 0.07). However, pre-treatment lysoGb3 values were comparable (p = 0.8).

### Relationship between biochemical response and clinical response

Figure 3c shows that the increase of plasma lysoGb3 levels after 1 year of shortage between males with and without antibodies to agalsidase beta did not differ significantly (8.6 vs. 5.7 nM; p = 0.4). Antibody titers did not influence the increase of lysoGb3 (data not shown). There was no correlation between baseline MSSI scores and increase in lysoGb3 (p = 0.8). Also, treatment duration or clinical event incidence before or after the shortage did not correlate to differences in lysoGb3 (p = 0.6, p = 0.8, p = 0.5).

## Discussion

The global enzyme shortage of agalsidase beta and the subsequent dose reductions caused distress and concern in both patients and physicians. This was further supported by an EMA report, describing an increase of reports of (serious) adverse events suggesting an accelerated course of Fabry disease [14]. We aimed to study the effects of the shortage by comparing clinical event incidence in the period before, to clinical event incidence during the shortage in the same patients. In summary, we did not find an increase in clinical event incidence during the agalsidase beta shortage or could establish that a reduced dose of agalsidase beta led to progression of disease during the shortage in an adult Fabry cohort. No definite conclusions can be drawn of the effect on left ventricular mass due to large variation of the (limited) data. During the shortage quality of life was significantly reduced in females on the vitality and general health subscales of the SF-36 questionnaire. The vitality subscale addresses energy level and tiredness and the general health perception subscale how patients experience their health (e.g. expectation of decreasing health). The SF-36 outcomes reported here are in accordance with earlier reports that demonstrated a decreased quality of life for male and female Fabry patients [26,27]. Because of the small sample size and wide confidence intervals, caution should be taken in interpreting these results, as the SF-36 differences before and during the shortage were rather small. It is unclear whether these differences in subscales actually represent a clinical meaningful detoriation, especially for the individual patient. This issue, also referred to as 'minimal clinical important difference' (MCID) is determined in many other chronic diseases. Comparing our mean changes to the minimal clinical important difference in the literature [21-23], it can be doubted whether the changes actually represent a clinical meaningful difference.

The difference in outcome of our study and the EMA report could be explained by several reasons. As mentioned by the EMA, reporting bias due to increased awareness for adverse events might have overestimated the number of adverse events reported. The advice of the EMA to reinstitute the recommended dose in patients with deterioration of the disease may have stimulated physicians to report adverse events, as this might have favoured allocation of agalsidase beta for their patients. In addition, some of the criteria used to suggest deterioration during the shortage were of rather subjective nature (e.g. fatigue, nausea, diarrhoea), but were nevertheless considered as an adverse event. It is questionable whether subjective criteria truly represent clinical detoriation. Finally, the EMA report used the Fabry registry to assess more objective criteria of clinical detoriation (occurrence of complications of the disease e.g. cardiovascular events). The incidence of these events was too low and the observational period too short to draw definite conclusions.

Importantly, we could establish an increase in plasma lysoGb3 in nearly all patients evaluated, following reduction of the therapeutic enzyme dose. Plasma lysoGb3 elevation is a hallmark of Fabry disease[28] and is associated with clinical manifestations[29]. Therefore, this observation leads to the conclusion that recurrence of storage material most likely has occurred in these patients. This is in line with observations made for lysoGb3 and Gb3 after induction of neutralizing antibodies [24,30,31]. We could not demonstrate a correlation between clinical event incidence and noted increases of lysoGb3. The lack of correlation with clinical outcome could be explained by the fact that enzyme replacement therapy has at best a modest effect on the course of the disease. In other words: complications will occur both in treated and untreated patients, ERT only results in a slightly different complication rate in patients with advanced disease [9,10] and at most stabilization of disease parameters in less severely affected patients [5,8]. Also, on the short term the expected number of clinical events of this slowly progressive disease would be rather low. Given the modest treatment effect, it is unlikely that on the short term a lower dose of enzyme therapy has a major impact on the incidence of complications in such a heterogeneous and small cohort. Therefore, on the short term the biochemical response is probably the most sensitive way to evaluate the effects of the shortage.

Interestingly, an increase in lysoGb3 occurred both in patients with an agalsidase beta reduction to 25% of the initial dose (1 mg/kg/eow) and those switched to agalsidase alpha (0.2 mg/kg/eow). We noted that the pre-shortage lysoGb3 values were higher in the *reduced agalsidase beta *group. One might argue that the expected increase of lysoGb3 in the *reduced agalsidase beta *group could be less pronounced than the *switch *group, because the pre-shortage values were already at their near maximum level (i.e. pre-treatment levels). However, as the pre-shortage samples in the *reduced agalsidase beta *group did nod reach pre-treatment values this is unlikely.

All patients with an available lysoGb3 value who switched to agalsidase alpha were previously dose reduced on agalsidase beta. Therefore, the increase in lysoGb3 could be attributed to the reduced agalsidase beta dose (and not to agalsidase alpha). However, these patients were approximately treated with agalsidase beta for six months, and subsequently with agalsidase alpha for about a year. It is therefore more likely that the increase is related to dose rather than preparation. This supports the earlier observations that not the enzyme preparation, but the doses are the most important determinants of biochemical response [24]. The importance of biochemical markers to assess the effects of dose changes is elegantly shown in Gaucher disease, where chitotriosidase reflects differences in doses [32] as well as impact of withdrawal of imiglucerase treatment, which coincides with clinically relevant deterioration of disease such as decreases in platelet counts [33-35].

There are several limitations to this study. The period in which we examined the outcome of the agalsidase beta shortage was rather short. It is possible that the incidence of clinical events increases over a longer observation period. Therefore, studies in larger cohorts, with a longer follow-up are required. Lastly, it remains difficult to determine whether the clinical events that occurred during the shortage-period are caused by the shortage itself, or by the progressive nature of the disease despite therapy[9]. The two clinical events that occurred within a month after the agalsidase beta shortage (an implantation of a pacemaker and a cardiac defibrillator) serve as an example: it is questionable (if not unlikely) whether these events were caused by a reduced agalsidase beta dose.

## Conclusion

In conclusion, we established no increase in the clinical event incidence during the shortage of agalsidase beta. We disagree with the conclusion from the EMA report of an accelerated course of disease as a result of a lower dose of Fabrazyme. The impact on the biochemical response, however, indicates the recurrence of storage material and supports the supposition that there is a biochemical dose effect of ERT.

## Competing interests

C.E.M. Hollak, G.E. Linthorst, J.M.F.G. Aerts received reimbursement of expenses and honoraria for lectures on the management of lysosomal storage diseases from Genzyme Corporation, Shire, Actelion and Amicus Therapeutics. All honoraria are donated to the Gaucher Stichting, a national foundation that supports research in the field of lysosomal storage disorders. J.M.F.G. Aerts has earlier received an unrestricted joint study grant from Genzyme Corporation and Shire HGT to investigate lysoGb3 in Fabry plasma specimens.

B.E. Smid and S.M. Rombach once received a travel support from Shire. The authors J.E. Groener, S. Kuiper, M. Mirzaian, H.S. Overkleeft, and B.J. Poorthuis, declare that they have no competing interests.

## Authors' contributions

BES performed acquisition, statistical analysis and interpretation of data, and drafting of the manuscript. GEL, CEMH, SMR participated in design of the study, interpretation of the data and helped to draft the manuscript. JEMG, MM and SK performed biochemical analyses and revised the manuscript. BJP, HSO, JMFGA developed a new method on tandem mass spectrometry to determine lysoGb3 and carefully revised the manuscript. All authors read and accepted the manuscript.
